# Over-the-Counter Naloxone and Nonprescription Syringe Availability in Community Pharmacies

**DOI:** 10.1001/jamanetworkopen.2024.58095

**Published:** 2025-02-03

**Authors:** Lindsey J. Loera, Jennifer E. Lines, Shannon R. Mayberry, Sarah A. Hilzendager, Aaron P. Ferguson, Lucas G. Hill

**Affiliations:** 1 College of Pharmacy, The University of Texas at Austin, Austin; 2 Texas Drug User Health Union, Austin

## Abstract

This cross-sectional study evaluates the accessibility of over-the-counter naloxone and nonprescription syringes in community pharmacies in Austin, Texas.

## Introduction

Community pharmacies can protect the health of people who use drugs (PWUD) by facilitating access to naloxone and syringes. Approval of some formulations of naloxone for over-the-counter (OTC) sale has the potential to advance these efforts. Studies before the OTC transition indicated naloxone was not consistently accessible, and little is known about stocking following this change.^1,2,3,4^ Limited available data indicate community pharmacists and technicians are often unwilling to sell nonprescription syringes.^5,6^

## Methods

This cross-sectional study is reported per Strengthening the Reporting of Observational Studies in Epidemiology (STROBE) guidelines and designed to evaluate the accessibility of OTC naloxone and nonprescription syringes in community pharmacies in Austin, Texas. The University of Texas at Austin institutional review board exempted the study from review and the need for informed consent. Every licensed community pharmacy (125 pharmacies) was visited in person by 1 of 5 trained auditors (J.E.L., S.R.M., S.A.H, and 2 volunteer student pharmacists) from February 10 to April 27, 2024. Auditors were student pharmacists who completed a 1-hour training in audit processes, a 1-hour training in harm reduction basics, and met with members of a statewide drug user union to prepare for the project. Each audit began with a visual inspection to evaluate OTC naloxone availability. The auditor then presented to the pharmacy counter and requested to purchase “a bag of 10 syringes” following a script described in a previous study.^6^ Auditors submitted research data immediately after each audit through a 10-item online form. Outcomes related to OTC naloxone included availability, cost, location in the pharmacy, and the presence of theft deterrent measures. Outcomes related to syringes included success of purchase attempts, cost, and reasons for unsuccessful attempts.

## Results

Data were obtained from all 125 pharmacies (100% response rate). A slight majority of pharmacies had a location for OTC naloxone (69 pharmacies [55.2%]) with all of these having a location for branded product but few also having a location for generic product (12 pharmacies [17.4%]). At least 1 physical box of OTC naloxone was in stock in 45 pharmacies with all of these having branded product available and 1 having generic available. The median (IQR) cost of the branded product ($44.99 [$44.97-$49.99]) was slightly higher than the generic product ($39.99 [$39.99-$39.99]). In 3 pharmacies where there was not a place on the OTC shelves for naloxone, auditors noted that branded product was visible behind the pharmacy counter. However, they were not able to confidently ascertain either the available stock or price. A slight majority of syringe purchase attempts were successful (73 attempts [58.4%]) and the median (IQR) cost was $3.19 ($4.00-$4.36). Pharmacy staff commonly asked clarifying or probing questions (115 [92.0%]) such as, “Why do you need them?,” and “Can I see your identification?”. The most common reason for syringe denial was requiring proof of a prescribed injectable medication (46 [88.5%]), such as insulin. Only 25 pharmacies (20.0%) would sell syringes and had at least 1 physical box of naloxone on an OTC shelf. Additional results related to OTC naloxone and nonprescription syringe availability are described in Tables 1 and 2.

**Table 1.** Over-the-Counter Naloxone Availability in 125 Pharmacies

Variable	Pharmacies, No. (%)		
	Any	Branded	Generic
Location on shelf for product	69 (55.2)	69 (55.2)	12 (17.4)
Theft deterrent measures^a^	35 (50.7)	35 (50.7)	6 (50.0)
Alarm sensor	17 (48.6)	17 (48.6)	1 (16.7)
Redeemable cards	11 (31.4)	11 (31.4)	3 (50.0)
Lock box	8 (22.9)	8 (22.9)	2 (33.3)
At least 1 physical box in stock	45 (65.2)	45 (65.2)	1 (8.3)
Available boxes of product, median (range)	4 (1-10)	4 (1-7)	10 (NA)
Cost, median (range), US $	44.99 (39.99-49.99)	44.99 (44.97-49.99)	39.99 (39.99-39.99)

Abbreviation: NA, not applicable.

^a^ The number of cases in which at least 1 theft deterrent measure was identified. This may be an underestimate given some measures are only identifiable when physical boxes are in stock.

**Table 2.** Nonprescription Syringe Availability in 125 Pharmacies

Variable	Pharmacies, No. (%)
Successful purchase	73 (58.4)
Cost, median (range), US $	3.19 (0-45.95)^a^
Reasons for denial	
Injectable medication prescription required	46 (88.5)
Pharmacy does not sell syringes	3 (5.8)
Syringes currently out of stock	3 (5.8)

^a^ The range reflects 2 pharmacies who offered a free 10-pack and 7 pharmacies who indicated they would only sell boxes of 100 syringes.

## Discussion

This is the first study we know of to rigorously evaluate the accessibility of OTC naloxone in US pharmacies following the approval of some formulations for OTC sale. Substantial gaps in accessibility and affordability of both OTC naloxone and nonprescription syringes were identified. OTC naloxone availability may have been undercounted if stored out of sight behind the counter and only available by request. Findings from 1 large city in the US South may not be generalizable to other contexts, and it is unknown how auditors being student pharmacists rather than people who inject drugs may have impacted the results. Efforts to educate pharmacy staff and support their implementation of evidence-based harm reduction strategies to safeguard the health of PWUD will be critical to addressing this public health emergency.

## Notes

Supplement 1.
Data Sharing Statement
