# Supplementary material for: Over-the-Counter Naloxone and Nonprescription Syringe Availability in Community Pharmacies
Source: JAMA Netw Open. 2025 Feb 3;8(2):e2458095. doi: 10.1001/jamanetworkopen.2024.58095 (PMC11791711; doi:10.1001/jamanetworkopen.2024.58095)
Supplement: Supplement 1. — Data Sharing Statement [file jamanetwopen-e2458095-s001.pdf]

Data Sharing Statement

Loera. Over-the-Counter Naloxone and Nonprescription Syringe Availability in Community Pharmacies. *JAMA Netw Open*. Published February 03, 2025. doi:10.1001/jamanetworkopen.2024.58095

Data
Data available: No
